# Compatibility of SYTO 13 and Hoechst 33342 for longitudinal imaging of neuron viability and cell death

**DOI:** 10.1186/1756-0500-5-437

**Published:** 2012-08-14

**Authors:** Kyle S Hubbard, Ian M Gut, Stephen M Scheeler, Megan E Lyman, Patrick M McNutt

**Affiliations:** 1 United States Army Medical Research Institute of Chemical Defense, 3100 Ricketts Point Rd, Aberdeen Proving Ground, MD, 21010, USA

**Keywords:** Nuclear staining, Pyknosis, Embryonic stem cell-derived neurons, Apoptosis, Necrosis

## Abstract

**Background:**

Simultaneous use of cell-permeant and impermeant fluorescent nuclear dyes is a common method to study cell viability and cell death progression. Although these assays are usually conducted as end-point studies, time-lapse imaging offers a powerful technique to distinguish temporal changes in cell viability at single-cell resolution. SYTO 13 and Hoechst 33342 are two commonly used cell-permeant nuclear dyes; however their suitability for live imaging has not been well characterized. We compare end-point assays with time-lapse imaging studies over a 6 h period to evaluate the compatibility of these two dyes with longitudinal imaging, using both control neurons and an apoptotic neuron model.

**Findings:**

In longitudinal assays of untreated neurons, SYTO 13 addition caused acute necrosis within 3 h, whereas neurons imaged with Hoechst remained viable for at least 6 h. In a staurosporine-induced apoptotic model of neurotoxicity, determinations of the mode of cell death and measurements of nuclear size were identical between longitudinal studies using Hoechst and end-point assays. Alternatively, longitudinal studies using 500 nM or 5 nM SYTO 13 were not consistent with end-point assays.

**Conclusions:**

SYTO 13 is acutely neurotoxic and when used in longitudinal studies, masked end-stage morphologic evidence of apoptotic cell death. In contrast, a single application of Hoechst evoked no evidence of toxicity over a 6 h period, and was consistent with end-point characterizations of cell viability and nuclear morphology. For longitudinal characterization of acute cell death, Hoechst is a superior option.

## Findings

### Background

The paired use of fluorescent membrane-permeant and impermeant nuclear dyes is an established method of evaluating morphological changes associated with end-stage cell death [1]. These dyes undergo a large fluorescent enhancement upon binding nucleic acids and, in conjunction with imaging methods such as flow cytometry or fluorescent microscopy, enable facile, rapid and quantitative discrimination between the classical terminal sequelae of apoptosis (condensed nuclei, intact membrane) and necrosis (normal or swollen nuclei, permeabilized plasma membrane) [2,3]. Cell-permeant nuclear markers such as SYTO 13 or Hoechst 33342 (Hoechst) provide information about nuclear morphology, while cells with disrupted plasma membranes are marked by nuclear uptake of cell-impermeant dyes such as propidium iodide (PI).

Nuclear markers are typically used in end-point assays to discriminate among viable, necrosed or apoptosed cells. When used in conjunction with live imaging, these dyes have the potential to enable longitudinal characterization of the onset of cell death with single-cell resolution. The statistical comparison of cell behavior using repeated measures over time allows for increased power and sensitivity compared to end-point assays, particularly in populations in which cell death occurs by multiple mechanisms. However, since acute cytotoxicity would confound interpretations of cell viability, membrane-permeant nuclear dyes have to be validated for longitudinal imaging. Here we evaluate the suitability of SYTO 13 and Hoechst for longitudinal imaging of neuron viability and identification of modes of end-stage cell death over a 6 h window.

### Baseline studies suggest SYTO 13 is incompatible with longitudinal cell viability

**Figure 1:**
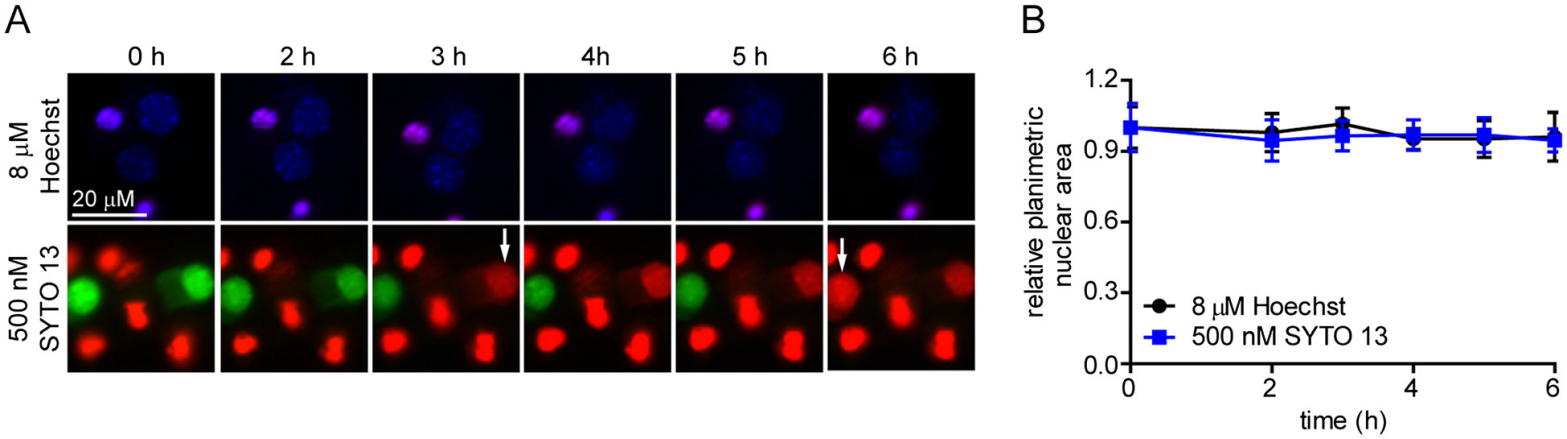
**Neurons visualized with SYTO 13 or Hoechst exhibit different outcomes during time-lapse microscopy. **(**A**) Neurons visualized with Hoescht (blue) remained viable over 6 h. Neurons stained with SYTO 13 (green) became PI-positive by 6 h, indicative of necrotic cell death. White arrows indicate cells that became PI-positive in each frame. (**B**) Repeated measures quantitation of nuclear area demonstrated no changes regardless of PI uptake.

In an attempt to develop a longitudinal assay of neuron viability, we evaluated nuclear morphology and plasma membrane integrity over a 6 h period after administration of 8 μM Hoechst or 500 nM SYTO 13. Unlike SYTO 13, which required hourly application, a single administration of Hoechst was sufficient to visualize nuclei throughout the 6 h experiment. Viable neurons were initially PI-negative, with nuclei that were 9.1 ± 0.66 μm in diameter. Whereas neurons exposed to Hoechst remained PI-negative through 6 h, neurons treated with SYTO 13 exhibited PI uptake within 3 h (Figure 1A). Neither dye elicited a significant change in nuclear size (Figure 1B). The co-localization of Hoechst and PI in neurons with disrupted membranes resulted in a purple hue (top panels), whereas the PI fluorescence overwhelmed SYTO 13 (bottom panel) unless the green channel gain was increased beyond the dynamic range (not shown). Condensed, PI-positive nuclei (purple in top panels, red in bottom panels) present at time 0 represent cells that underwent primary apoptosis during initial plating followed by secondary necrosis, a common phenomenon with *in vitro* models [4].

### Longitudinal use of SYTO 13 alters neuron cell death in a model of apoptosis

The above experiments suggested that SYTO 13 was acutely neurotoxic. To determine whether the presumptive toxicity associated with SYTO 13 interfered with morphological determination of apoptotic cell death, we evaluated nuclear morphology and plasma membrane integrity among neurons treated with the pro-apoptotic kinase inhibitor staurosporine [5]. Neuron fate was compared between end-point assays conducted 6 h after staurosporine addition and longitudinal assays, in which nuclear morphologies were imaged every hour for 6 h using PI with either SYTO 13 or Hoechst uptake.

**Figure 2:**
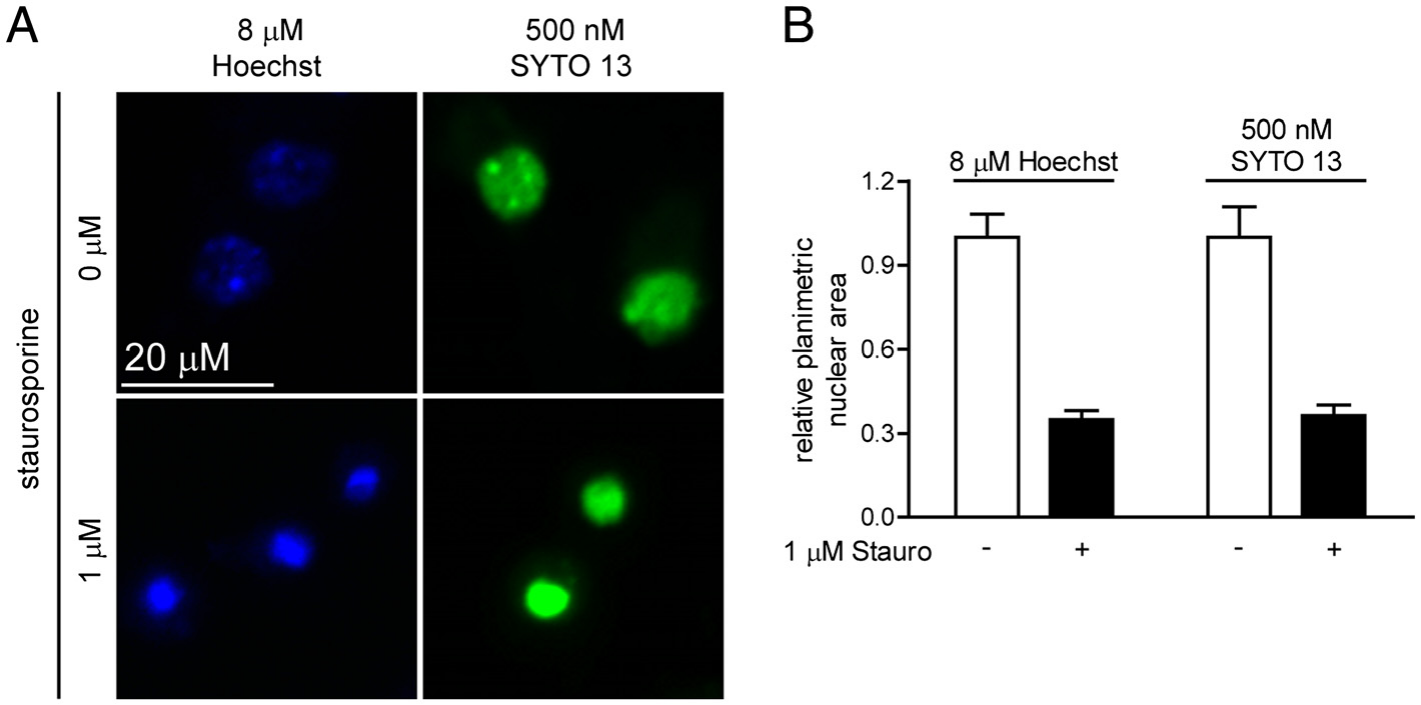
**End-point assays of control and staurosporine-treated neurons exhibit identical outcomes at 6 h. **(**A**) End-point characterization of nuclear morphology 6 h after addition of vehicle (top panels) or staurosporine (bottom panels). Neurons were visualized using PI plus either Hoescht (blue) or SYTO 13 (green). Note that neither vehicle nor staurosporine addition caused necrotic cell death. (**B**) Planimetric measurements of nuclear size. * represent p < 0.01.

**Figure 3:**
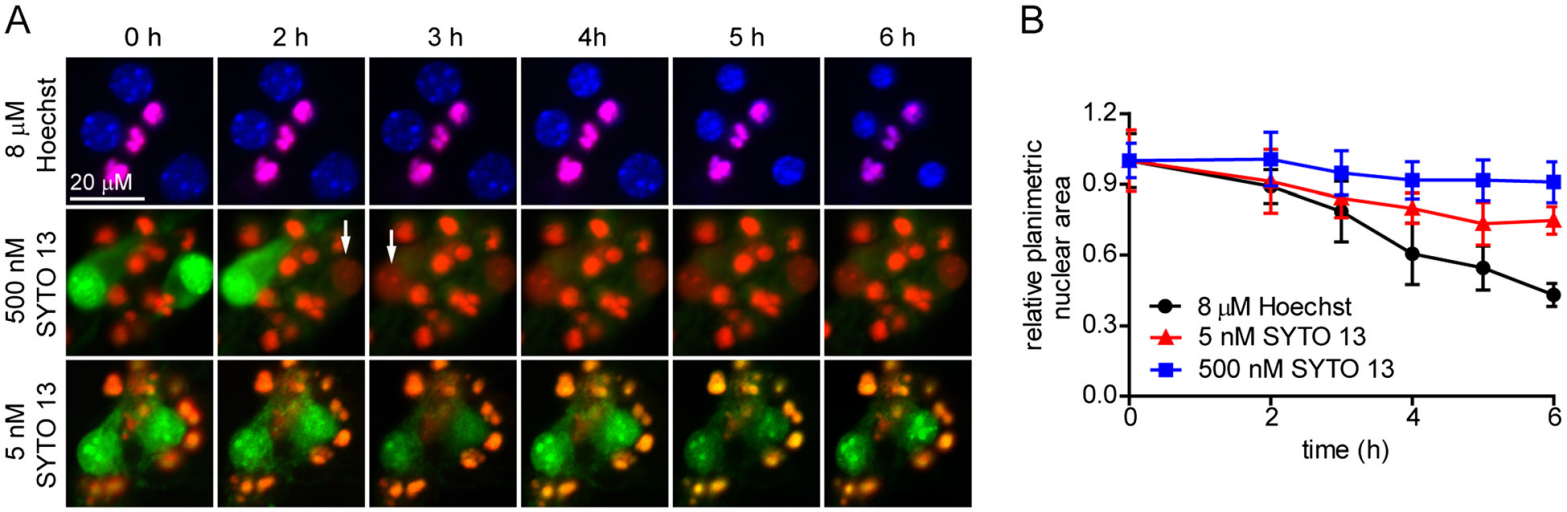
**Staurosporine-treated neurons undergo different outcomes when SYTO 13 and Hoechst are used in time-lapse imaging. **(**A**) Neurons stained with Hoescht (blue; top panels) visually undergo chromatin condensation within 4 h and remained PI-negative. In contrast, neurons visualized with 500 or 5 nM SYTO 13 (green) became PI-positive (red) by 2 h, without visual evidence of chromatin condensation. White arrows indicate cells that became PI-positive in each frame. Condensed, PI-positive nuclei (purple in top panels, red in middle and bottom panels) observed at time 0 represent cells that died during initial plating. In bottom panels, the increased gain needed to clearly image nuclear structure resulted in PI-positive nuclei developing an orange hue. (**B**) Planimetric quantitation of nuclear size measured longitudinally through the 6 h experiment. * represent p < 0.05; ^ represents p < 0.01.

In end-point assays conducted 6 h after staurosporine addition, neurons had PI-negative, condensed nuclei that were 35.0 ± 5.1% (Hoechst) or 37.3 ± 7.4% (SYTO 13) the area of untreated controls (Figure 2; *P* < 0.001 for both dyes). In longitudinal imaging using Hoechst, nuclear condensation was visually apparent by 4 h, with PI-negative nuclei at 6 h that were 41.1 ± 6.4% of the area measured at 0 h (Figure 3; *P* < 0.001). In contrast, SYTO 13-stained neurons were PI-positive by 2 h, without evidence of nuclear condensation through 6 h (Figure 3). As in Figure 1, the PI signal overwhelmed the SYTO 13 signal at 500 nM.

These data unambiguously demonstrate that neurons exposed to repeated doses of 500 nM SYTO 13 underwent acute necrosis, even when exposed to apoptotic stimuli. Reasoning that SYTO 13 neurotoxicity may be dose-dependent, neurons were evaluated by time-lapse imaging using 5 nM SYTO 13. The lower concentration of SYTO 13 eliminated PI uptake in staurosporine-treated neurons throughout 6 h (Figure 3A). Nuclear diameters were difficult to measure because of diffuse nuclear staining; however, at the lower SYTO 13 dose nuclear areas were only reduced to 74.7 ± 12.9% relative to 0 h (Figure 3B).

## Discussion

Defining the molecular and biochemical pathways responsible for cell death phenotypes is essential for identifying critical points that could be exploited to prevent, delay or re-direct cell death mechanisms. This requires the ability to conduct time-lapse characterization of multiple aspects of the onset and progression of cell death. However, a longitudinal assay to visualize the morphologic alterations associated with cell death has not been well-described. Here we demonstrate that SYTO 13 is acutely neurotoxic *in vitro*, whereas Hoescht-treated neurons do not exhibit symptoms of toxicity over a 6 h period. Thus for short-term longitudinal imaging of viability and morphologic characterization of apoptotic cell death Hoechst is the preferred dye.

Despite manufacturer warnings that SYTO 13 is not recommended for imaging nuclear morphology, a number of publications have used SYTO 13 to evaluate nuclear changes associated with cell death phenotypes, in some cases during longitudinal cell viability assays [6-9]. Here we demonstrate that 500 nM SYTO 13 elicited acute necrosis and prevented staurosporine-induced chromatin condensation in time-lapse imaging studies. Reduction of SYTO 13 to 5 nM prevented acute necrosis, but resulted in less intense nuclear staining and an intermediate degree of nuclear condensation compared to end-point studies. In contrast, control neurons longitudinally imaged with Hoechst exhibited no morphological evidence of neurotoxicity over 6 h, and underwent a similar degree of chromatin condensation following staurosporine treatment as in end-point studies.

The mechanism(s) of the acute SYTO 13 neurotoxicity described here is unclear, but could be a consequence of nuclear binding or interaction with cytoplasmic nucleic acids. Regardless of the mechanism, the end-stage cell death markers of necrosis were observed prior to those of apoptosis, suggesting that acute neurotoxicity interferes with apoptotic progression. There are several possible explanations for the intermediate degree of chromatin condensation observed with the lower dose of SYTO 13. For example, injury mechanisms may be initiated that do not result in overt necrosis by 6 h, yet deprive the cell of sufficient metabolic energy to delay apoptotic progression. Alternatively, SYTO 13 may sterically interfere with the mechanisms involved in chromosome nicking and chromatin condensation, disrupting the morphological appearance of end-stage apoptosis.

Hoechst is a superior alternative to SYTO 13 for reasons other than the low relative toxicity. SYTO 13 labels cytoplasmic RNA as well as nuclear DNA, complicating measurements of nuclear size. Hoechst, on the other hand, preferentially intercalates between adenine and thymine in DNA minor grooves, rendering it specific for nuclear chromatin. Unlike SYTO 13, which had to be applied on an hourly basis, a single application of Hoechst was sufficient for 6 h, significantly reducing reagent costs and the potential for cumulative toxicity. Although reducing the concentration of SYTO 13 decreased the intensity of non-nuclear fluorescence, the weak nuclear signal made planimetric measurements difficult.

## Conclusions

In a comparative evaluation of the suitability of SYTO 13 and Hoechst for time-lapse imaging studies of neuron viability and cell death, we found that SYTO 13 was acutely neurotoxic at high doses within 3 h, whereas Hoechst-treated neurons remained viable for at least 6 h. In an apoptotic model, 5 and 500 nM SYTO 13 interfered with the ability to distinguish between apoptotic and necrotic cell death. In contrast, a single administration of 8 μM Hoechst was sufficient to identify nuclear condensation by 4 h, without evidence of necrosis through 6 h. Neurons are generally sensitive to external insults, and thus may be more susceptible to SYTO 13 than other cell lines. Nonetheless, given the evidence of acute toxicity in SYTO 13-treated cultures, Hoechst is a superior alternative for short-term, longitudinal evaluations of neuron viability and nuclear morphology.

## Methods

### Embryonic stem cell culture and neuronal differentiation

ESNs were generated, plated and cultured as previously described [10,11]. ESNs were maintained in Neurobasal®-A medium (NBA, Life Technologies, Carlsbad, CA) with glutamine, B27 supplement and penicillin/streptomycin (Sigma-Aldrich, St Louis, MO) at 37°C and 5% CO_2_.

### Morphological evaluation of DNA condensation and membrane disruption

For longitudinal imaging, DIV 14 ESNs plated on 18 mm coverslips were transferred to a Zeiss LSM-700 confocal microscope stage in a constant-temperature environmental chamber at 37°C and incubated in Hibernate®-E, media supplemented with glutamine, B27 vitamins and penicillin/streptomycin (Life Technologies), PI (10 μg/mL, Sigma-Aldrich) and either SYTO 13 (500 nM or 5 nM, Life Technologies) or Hoechst (5 μg/mL, (Intergen, Oxford, U.K.) for 10 minutes. After the incubation period, neurons were perfused with complete Hibernate®-E medium using a Minipuls 3 peristaltic pump (Gilson, Inc) at 200 μL/minute. After 50 minutes, the pump was turned off and PI (and SYTO 13, if used) was added to the coverslip for 10 minutes. The pump was then restarted and images were captured at 20x using manufacturer-specified excitation wavelengths and emission filter sets. These steps were repeated on an hourly basis for 6 h. For a neuronal model of apoptosis, staurosporine (Sigma-Aldrich) was applied at 1 μM in the perfusion media throughout the 6 h experiment.

For end-point assays, ESNs on 18-mm coverslips in 12-well dishes were washed and incubated for 6 h in complete NBA medium without (control) or with 1 μM staurosporine. After 6 h, the media was replaced with complete NBA supplemented with 5 μg/mL PI for 10 min at 37°C and 5% CO_2_ followed by complete NBA with 5 μg/mL Hoechst or 500 nM SYTO 13 for 5 min. Coverslips were then washed twice with PBS, fixed in 4% paraformaldehyde in PBS for 20 min, mounted on glass slides with Prolong Gold antifade reagent and imaged as above.

### Planimetric measurements of nuclear area

Twenty nuclei per time point were measured along orthogonal axes using Zen 2009 and averaged. The same nuclei were measured longitudinally from 0–6 h. A similar measurement methodology was used for end-point assays, except over 50 nuclei. Average diameters were converted to areas and graphed with Prism version 5.04 (GraphPad Software). Longitudinal changes in mean nuclear sizes were compared using repeated-measures ANOVA and significances determined with Tukey’s post-hoc comparison. Student’s t-tests were used to compare the mean nuclear sizes in end-point assays.

## Abbreviations

PI: propidium iodide; ESNs: embryonic stem cell-derived neurons; Hoechst: Hoechst 32444; NBA: Neurobasal-A medium.

## Competing interests

The authors declare that they have no competing interests.

## Authors’ contributions

KSH conceived of the study, designed the study, executed the live imaging, interpreted the results and contributed to the manuscript. IMG contributed to the study design, interpreted results, conducted the end point assays and prepared figures for the manuscript. SMM conducted live imaging and interpreted results. MLL maintained and differentiated neurons and contributed to the manuscript. PMM conceived of the study, designed the study, interpreted the results and drafted the manuscript. All authors have read and approved the final version of the manuscript.

## Funding information

This work was funded by the Defense Threat Reduction Agency – Joint Science and Technology Office, Medical S&T Division (grant number CBM. THRTOX.01.10.RC.021). I.G. was supported by a Defense Threat Reduction Agency-National Research Council Research Associateship Award.
